# First-Line Palbociclib/Letrozole Versus Ribociclib/Letrozole in Postmenopausal HR-Positive/HER2-Negative Metastatic Breast Cancer: A Retrospective Real-World Study and an Exploratory Composite Clinical-Inflammatory Risk Score

**DOI:** 10.3390/medicina62081470

**Published:** 2026-07-29

**Authors:** Mert Tohumcuoğlu, Abdullah Evren Yetişir, Zafer Ufuk Cinkara, Mehmet Türker, Alpay Düşgün, Cem Mirili, Deniz Tazeoğlu, Tolga Köşeci, Ali Oğul, Mahmut Büyükşimşek

**Affiliations:** 1 Department of Medical Oncology, Adana City Training and Research Hospital, University of Health Sciences, Adana 01230, Türkiye; evrenyetisir@hotmail.com (A.E.Y.); ufukcinkara@hotmail.com (Z.U.C.); mmtturker@hotmail.com (M.T.); alpaydusgun@hotmail.com (A.D.); mahmutbuyuksimsek@gmail.com (M.B.); 2 Medical Oncology Clinic, Adana Ortadoğu Hospital, Adana 01140, Türkiye; cemirili@gmail.com; 3 Department of Surgical Oncology, Osmaniye State Hospital, Osmaniye 80010, Türkiye; deniztazeoglu@gmail.com; 4 Department of Medical Oncology, Faculty of Medicine, Çukurova University, Adana 01330, Türkiye; drtolgakoseci@gmail.com; 5 Department of Medical Oncology, Faculty of Medicine, Mersin University, Mersin 33343, Türkiye; mdaliogul@gmail.com

**Keywords:** hormone receptor-positive/HER2-negative metastatic breast cancer, palbociclib, ribociclib, CDK4/6 inhibitor, pan-immune-inflammation value, progesterone receptor, visceral metastasis, composite risk score

## Abstract

*Background and Objectives*: Palbociclib and ribociclib are widely used with letrozole as first-line treatment for postmenopausal patients with hormone receptor-positive/human epidermal growth factor receptor 2-negative metastatic breast cancer. Pivotal trials have reported different overall survival signals, but they were not designed as direct comparisons. This study evaluated real-world survival outcomes with these regimens and explored the prognostic performance of a composite clinical-inflammatory risk score. *Materials and Methods*: This retrospective single-center cohort included 140 postmenopausal patients treated with first-line palbociclib/letrozole or ribociclib/letrozole. Overall survival, progression-free survival, best documented response, baseline characteristics, metastatic pattern, and baseline pan-immune-inflammation value were evaluated. The composite risk score assigned one point each for progesterone receptor expression <20%, visceral metastasis, and pan-immune-inflammation value ≥477.8. Pan-immune-inflammation value was additionally evaluated as a continuous variable. The prognostic performance of the score was assessed using Harrell’s c-index, bootstrap internal validation, and comparison with a parsimonious clinical model. *Results*: Among 140 patients, 67 received palbociclib/letrozole and 73 received ribociclib/letrozole. Median follow-up was 52.3 months. A total of 93 deaths and 118 progression-free survival events occurred. Median overall survival was 36 months with palbociclib/letrozole and 29 months with ribociclib/letrozole, with no statistically significant difference between groups (HR, 1.23; 95% CI, 0.82–1.86; *p* = 0.322). Median progression-free survival was 15.4 and 16.4 months, respectively (HR, 1.05; 95% CI, 0.73–1.52; *p* = 0.778). Median overall survival was 34 months for CRS 0–1 and 30 months for CRS 2–3 (univariable HR, 1.68; 95% CI, 1.11–2.54; *p* = 0.014). The apparent and optimism-corrected c-indices of the score were 0.573 and 0.570, respectively. Addition of the score to the parsimonious clinical model did not significantly improve model fit (likelihood-ratio *p* = 0.143). *Conclusions*: No statistically detectable difference in overall survival or progression-free survival between the treatment groups was observed in this cohort. Although grouped CRS was associated with overall survival in univariable analysis, the score showed limited discrimination and no statistically significant incremental prognostic value. These findings remain exploratory and require external validation.

## 1. Introduction

Hormone receptor-positive/HER2-negative breast cancer is the most common biological subtype of breast cancer and accounts for a large proportion of patients with metastatic disease. In this setting, endocrine therapy remains the main treatment backbone, particularly in patients without visceral crisis. However, endocrine therapy alone is often not sufficient. Some tumors show primary endocrine resistance, while others initially respond and later progress because of acquired resistance. For this reason, the addition of cyclin-dependent kinase 4/6 inhibitors to endocrine therapy has become a standard first-line treatment approach for patients with HR-positive/HER2-negative metastatic breast cancer who are suitable for endocrine-based treatment [1,2].

Palbociclib and ribociclib are widely used CDK4/6 inhibitors in combination with aromatase inhibitors. In PALOMA-2, palbociclib plus letrozole significantly improved progression-free survival compared with placebo plus letrozole in postmenopausal patients with ER-positive/HER2-negative advanced breast cancer [3]. In the final overall survival analysis of PALOMA-2, however, palbociclib plus letrozole did not show a statistically significant OS advantage over letrozole alone, and this result was affected by missing survival data and post-progression treatment patterns [4]. In contrast, MONALEESA-2 showed a significant OS benefit with ribociclib plus letrozole in a first-line postmenopausal population [5]. OS benefit with ribociclib-based endocrine therapy was also reported in MONALEESA-7 and MONALEESA-3, supporting the consistency of ribociclib data across different patient groups and endocrine therapy partners [6,7].

These trial-level differences are clinically relevant, but they should not be interpreted as a direct comparison between palbociclib and ribociclib. PALOMA-2 and MONALEESA-2 were separate trials, with differences in design, follow-up, subsequent therapies, patient characteristics, and missing data patterns. More importantly, there is no randomized head-to-head trial directly comparing palbociclib and ribociclib in the first-line metastatic setting. In routine practice, the choice between these agents is shaped by several factors, including comorbidities, baseline blood counts, cardiac risk, liver function, drug interactions, toxicity profile, reimbursement conditions, and physician preference.

Real-world comparative data are therefore useful, especially when they include patients who are less selected than those enrolled in pivotal trials. Available real-world studies are not completely uniform. The OPAL registry reported no clear difference in progression-free survival or overall survival between first-line palbociclib and ribociclib, whereas PALMARES-2 suggested that outcomes may differ among CDK4/6 inhibitors in selected real-world populations [8,9]. These differences may be related to baseline risk, endocrine sensitivity, metastatic burden, treatment exposure, dose modification patterns, follow-up duration, and local treatment practice. Additional real-world cohorts are therefore still relevant, particularly when treatment outcomes are interpreted together with clinical and biological risk factors rather than as a simple drug-versus-drug comparison.

Patients with HR-positive/HER2-negative metastatic breast cancer also show considerable prognostic heterogeneity, even when treated with first-line CDK4/6 inhibitor plus endocrine therapy. Some patients achieve prolonged disease control, whereas others experience early progression or shorter survival. Traditional clinical and pathological variables, including visceral involvement, disease burden, tumor grade, and hormone receptor expression, remain useful in estimating baseline risk. Among these variables, progesterone receptor expression may be particularly informative. Low or absent PR expression is generally considered a marker of less endocrine-sensitive tumor biology, and recent CDK4/6 inhibitor-treated cohorts have suggested an association between low PR expression and poorer outcomes [10,11].

Systemic inflammation is another clinically accessible area of interest in metastatic breast cancer. Inflammation-based indices are not specific to breast cancer, but they may reflect the interaction between tumor burden, host immune response, and the systemic inflammatory state. The pan-immune-inflammation value (PIV), calculated from neutrophil, platelet, monocyte, and lymphocyte counts, combines several blood-based inflammatory parameters into a single index [12]. PIV has been evaluated in different malignancies, and available breast cancer data suggest that higher PIV may be associated with poorer survival outcomes [13]. Its practical advantage is that it can be calculated from routine complete blood count parameters without additional testing.

Still, a single inflammatory marker is unlikely to capture the full clinical risk of patients with metastatic HR-positive/HER2-negative breast cancer. Combining a blood-based inflammatory index with routinely available clinical and pathological variables may offer an exploratory approach to baseline prognostic stratification. In this context, a composite risk score incorporating PIV, progesterone receptor expression, and visceral metastasis warrants investigation in patients treated with first-line CDK4/6 inhibitor plus endocrine therapy. We considered the score exploratory and intended it for hypothesis generation rather than clinical decision-making.

In this retrospective real-world cohort, we evaluated survival outcomes with first-line palbociclib/letrozole and ribociclib/letrozole in postmenopausal patients with HR-positive/HER2-negative metastatic breast cancer. We also examined whether an exploratory composite risk score based on progesterone receptor expression, visceral metastasis, and PIV could stratify overall survival in this patient population.

## 2. Materials and Methods

### 2.1. Study Design and Patient Population

This retrospective cohort study was conducted at the Department of Medical Oncology, Adana City Training and Research Hospital, Adana, Türkiye. The study included postmenopausal patients with hormone receptor-positive/HER2-negative metastatic breast cancer who received first-line CDK4/6 inhibitor plus letrozole therapy. Patients who started treatment with palbociclib/letrozole or ribociclib/letrozole between February 2020 and May 2025 were screened for eligibility. Follow-up data were updated in January 2026.

**Figure 1:**
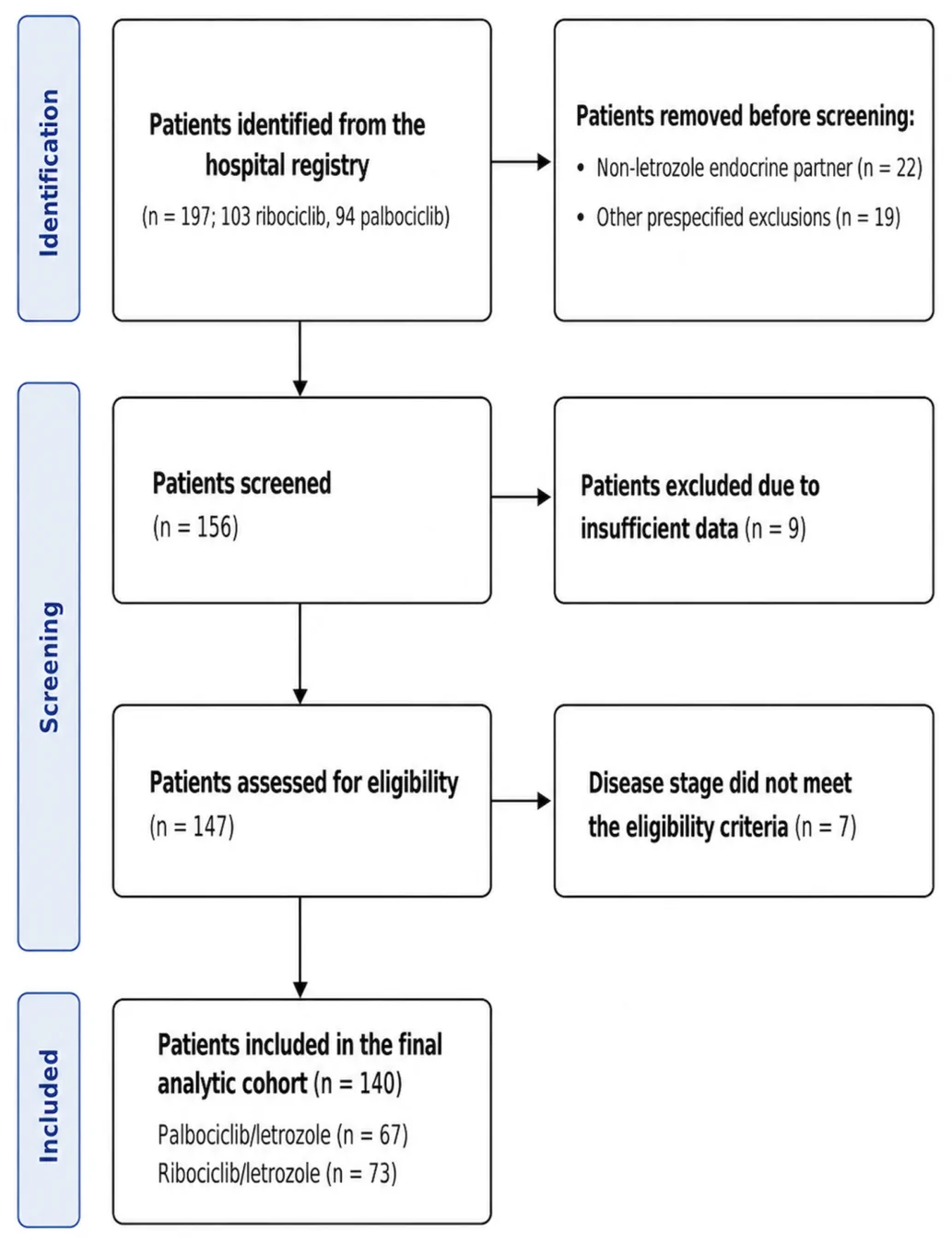
Flow diagram of patient identification, screening, eligibility assessment, and inclusion from the hospital registry.

Patient selection is summarized in Figure 1. A total of 197 patient records were identified from the hospital registry. Forty-one records were excluded before screening, including 22 patients who received a non-letrozole endocrine partner and 19 who met other prespecified exclusion criteria. Among 156 patients screened, 9 were excluded because of insufficient data. Of the remaining 147 patients assessed for eligibility, seven additional patients were excluded because their disease stage did not meet the eligibility criteria. The final analytic cohort consisted of 140 patients, including 67 treated with palbociclib/letrozole and 73 treated with ribociclib/letrozole.

### 2.2. Eligibility Criteria

Patients were included if they were 18 years of age or older, postmenopausal, had histologically confirmed hormone receptor-positive/HER2-negative metastatic breast cancer, and received palbociclib plus letrozole or ribociclib plus letrozole as first-line treatment for metastatic disease. Availability of baseline clinicopathologic, laboratory, treatment, response, and follow-up data was also required. Patients were excluded if they had HER2-positive disease, received a first-line metastatic regimen other than palbociclib/letrozole or ribociclib/letrozole, had insufficient clinical or follow-up data, had no evaluable follow-up after treatment initiation, or had inconsistent diagnosis, treatment sequence, or outcome records after detailed review.

### 2.3. Treatment Groups

Patients were grouped according to the CDK4/6 inhibitor used in combination with letrozole. Palbociclib was generally administered at a starting dose of 125 mg once daily on days 1–21 of a 28-day cycle. Ribociclib was generally administered at a starting dose of 600 mg once daily on days 1–21 of a 28-day cycle. Letrozole was given orally at a dose of 2.5 mg once daily in both groups. Dose modifications or treatment interruptions were made at the treating physician’s discretion, mainly according to toxicity and clinical status. Treatment was continued until disease progression, unacceptable toxicity, death, or physician decision.

### 2.4. Data Collection and Variables

Clinical, pathological, laboratory, treatment, and follow-up data were obtained from institutional electronic medical records and patient files. The recorded variables included age at CDK4/6 inhibitor initiation, ECOG performance status, estrogen receptor status, progesterone receptor status, Ki-67 index, histologic grade, metastatic presentation, metastatic sites, number of metastatic organs, baseline complete blood count parameters, treatment group, best documented response, progression status, vital status, and dates of treatment initiation, progression, last follow-up, and death. Hormone receptor and HER2 status were determined from pathology reports based on routine immunohistochemical assessment, with in situ hybridization used when clinically indicated. Metastatic presentation was categorized as de novo metastatic or recurrent disease. Metastatic sites were recorded as bone, distant lymph node, and visceral involvement. Visceral metastasis was defined as involvement of the liver, lung, pleura/malignant pleural effusion, peritoneum/carcinomatosis, brain/CNS, or other visceral organs.

### 2.5. Calculation of Pan-Immune-Inflammation Value

Baseline laboratory values were defined as the closest available complete blood count values obtained before CDK4/6 inhibitor initiation. Neutrophil, platelet, monocyte, and lymphocyte counts were used to calculate the pan-immune-inflammation value (PIV) as follows:

PIV = neutrophil count × platelet count × monocyte count/lymphocyte count

Receiver operating characteristic curve analysis was used to determine PIV cutoff values for progression and death. Cutoff values were selected using Youden’s J index. The cutoff derived for death was used to define elevated PIV in the composite risk score.

### 2.6. Definition of Composite Risk Score

A composite risk score was constructed using three baseline variables that were routinely available at treatment initiation: progesterone receptor expression, visceral metastasis, and PIV. One point was assigned for each adverse factor: progesterone receptor expression < 20%, presence of visceral metastasis, and PIV ≥ 477.8. The total CRS ranged from 0 to 3. The 20% PR threshold was used to distinguish absent or low PR expression from preserved PR expression as a clinically interpretable marker of reduced endocrine sensitivity. Because only five patients had CRS 3, survival analyses focused on the comparison between patients with CRS 0–1 and those with CRS 2–3. The four individual score categories were retained for descriptive presentation only.

### 2.7. Outcome Assessment

The survival endpoints were overall survival and progression-free survival. Overall survival was defined as the time from initiation of palbociclib/letrozole or ribociclib/letrozole to death from any cause or the date of last follow-up. Patients who were alive at the last follow-up were censored. Progression-free survival was defined as the time from treatment initiation to documented disease progression, death, or last follow-up, whichever occurred first. Patients without progression or death at the last follow-up were censored.

Best documented response was retrospectively abstracted from available routine-care radiologic reports and clinical records and categorized as complete response, partial response, stable disease, or progressive disease. Response assessment was not based on prospective, protocol-mandated, centrally reviewed RECIST evaluation. Objective response rate was defined as the proportion of patients with complete or partial response. Disease control rate was defined as the proportion of patients with complete response, partial response, or stable disease as the best documented response.

### 2.8. Statistical Analysis

All descriptive, comparative, survival, proportional hazards diagnostics, c-index, and bootstrap internal validation analyses were performed using IBM SPSS Statistics, version 25.0 (IBM Corp., Armonk, NY, USA). Normality of continuous variables was assessed using the Shapiro–Wilk test. Continuous variables were presented as median and interquartile range, and categorical variables were presented as frequencies and percentages. Continuous variables were compared between treatment groups using the Mann–Whitney U test. Categorical variables were compared using the chi-square test, Fisher’s exact test, or Fisher–Freeman–Halton exact test, as appropriate. The distribution of treatment initiation year between treatment groups was compared using the Fisher–Freeman–Halton exact test. Receiver operating characteristic curve analysis was used to evaluate the ability of baseline PIV to discriminate progression and death. Cutoff values were selected using Youden’s J index. Because the cutoff for death was derived and evaluated in the same cohort, analyses based on this threshold were regarded as exploratory.

Survival curves were estimated using the Kaplan–Meier method and compared using the log-rank test. Median follow-up was estimated using the reverse Kaplan–Meier method. Cox proportional hazards regression was used to estimate hazard ratios and 95% confidence intervals. The proportional hazards assumption was evaluated using tests based on Schoenfeld residuals.

In addition to analysis using the ROC-derived categorical cutoff, PIV was modeled continuously per one-standard-deviation increase and after log_2_ transformation, with the latter expressed per doubling.

The grouped CRS survival analysis compared CRS 0–1 with CRS 2–3. The discriminative performance of CRS was evaluated using Harrell’s c-index. Optimism correction for CRS alone was estimated using 2000 bootstrap resamples. To evaluate the incremental prognostic contribution of CRS, a parsimonious clinical Cox model including histologic grade, de novo versus recurrent metastatic presentation, number of metastatic organs, and treatment group was compared with an expanded model additionally including CRS as an ordinal variable. Model complexity was deliberately restricted, and ECOG performance status was not included; therefore, residual confounding related to performance status cannot be excluded. Model comparison and the bootstrap interval for the change in c-index were based on 1000 bootstrap resamples. Nested models were compared using a likelihood-ratio test.

All tests were two-sided, and *p* values < 0.05 were considered statistically significant. Patients with missing or incomplete required clinical, laboratory, treatment, response, or follow-up data were excluded from the final analytic cohort. Accordingly, a complete-case analysis was performed, and no imputation was used.

### 2.9. Ethics Committee Approval

The study protocol was reviewed and approved by the Scientific Research Ethics Committee of Adana City Training and Research Hospital, Adana, Türkiye (Meeting No: 17; Date: 25 September 2025; Decision No: 760). The study was conducted in accordance with the principles of the Declaration of Helsinki. The requirement for written informed consent was waived because of the retrospective design of the study and the use of anonymized patient data.

## 3. Results

### 3.1. Baseline Characteristics and Treatment Response

Patient selection is shown in Figure 1. Of 197 records identified from the hospital registry, 140 patients were included in the final analytic cohort after the exclusion process. The final cohort included 67 patients treated with palbociclib/letrozole and 73 treated with ribociclib/letrozole as first-line CDK4/6 inhibitor-based therapy. The median age of the overall cohort was 60 years. Age was similar between the palbociclib/letrozole and ribociclib/letrozole groups (59 vs. 61 years, respectively; *p* = 0.923).

Baseline clinical and pathological characteristics according to treatment group are shown in Table 1. ECOG performance status, Ki-67 index, histologic grade, metastatic presentation, bone metastasis, distant lymph node metastasis, and visceral metastasis did not differ significantly between the two treatment groups. All patients had estrogen receptor-positive disease. Progesterone receptor status, categorized as negative, low, or high expression, was also not significantly different between groups (*p* = 0.088). The median baseline pan-immune-inflammation value (PIV) was 362.0 in the overall cohort. PIV was higher in the palbociclib/letrozole group than in the ribociclib/letrozole group (393.1 vs. 328.4; *p* = 0.015).

De novo metastatic disease was present in 57 patients (40.7%), whereas 83 patients (59.3%) had recurrent metastatic disease. Bone metastasis was the most common metastatic site and was observed in 116 patients (82.9%). Bone-only disease was present in 55 patients (39.3%), distant lymph node metastasis in 46 patients (32.9%), and visceral metastasis in 66 patients (47.1%). Visceral metastasis was numerically more frequent in the ribociclib/letrozole group than in the palbociclib/letrozole group, but the difference was not statistically significant (52.1% vs. 41.8%; *p* = 0.224). The number of metastatic organs differed between treatment groups (*p* = 0.046). Single-organ metastatic disease was more common in the palbociclib/letrozole group, whereas two-organ metastatic disease was more common in the ribociclib/letrozole group.

Treatment initiation year differed significantly between groups (Fisher–Freeman–Halton exact *p* < 0.001). Palbociclib use was concentrated in 2020–2021, during which 46 of 67 patients (68.7%) initiated treatment, whereas ribociclib use was concentrated in 2022–2024, during which 51 of 73 patients (69.9%) initiated treatment. The median treatment initiation dates were 14 July 2021 in the palbociclib/letrozole group and 16 January 2023 in the ribociclib/letrozole group.

Treatment response data are also summarized in Table 1. The distribution of best documented response differed between groups (*p* = 0.029). The largest absolute between-group differences were observed in the stable-disease and progressive-disease categories. In the overall cohort, complete response was documented in 5 patients (3.6%), partial response in 104 patients (74.3%), stable disease in 15 patients (10.7%), and progressive disease in 16 patients (11.4%). However, neither ORR nor DCR differed significantly between treatment groups. ORR was 82.1% with palbociclib/letrozole and 73.9% with ribociclib/letrozole (*p* = 0.248), whereas DCR was 85.1% and 91.8%, respectively (*p* = 0.213). Accordingly, the difference in the overall response distribution was not interpreted as evidence of superior response efficacy for either treatment.

### 3.2. ROC Analysis of PIV and Distribution of Composite Risk Score

**Figure 2:**
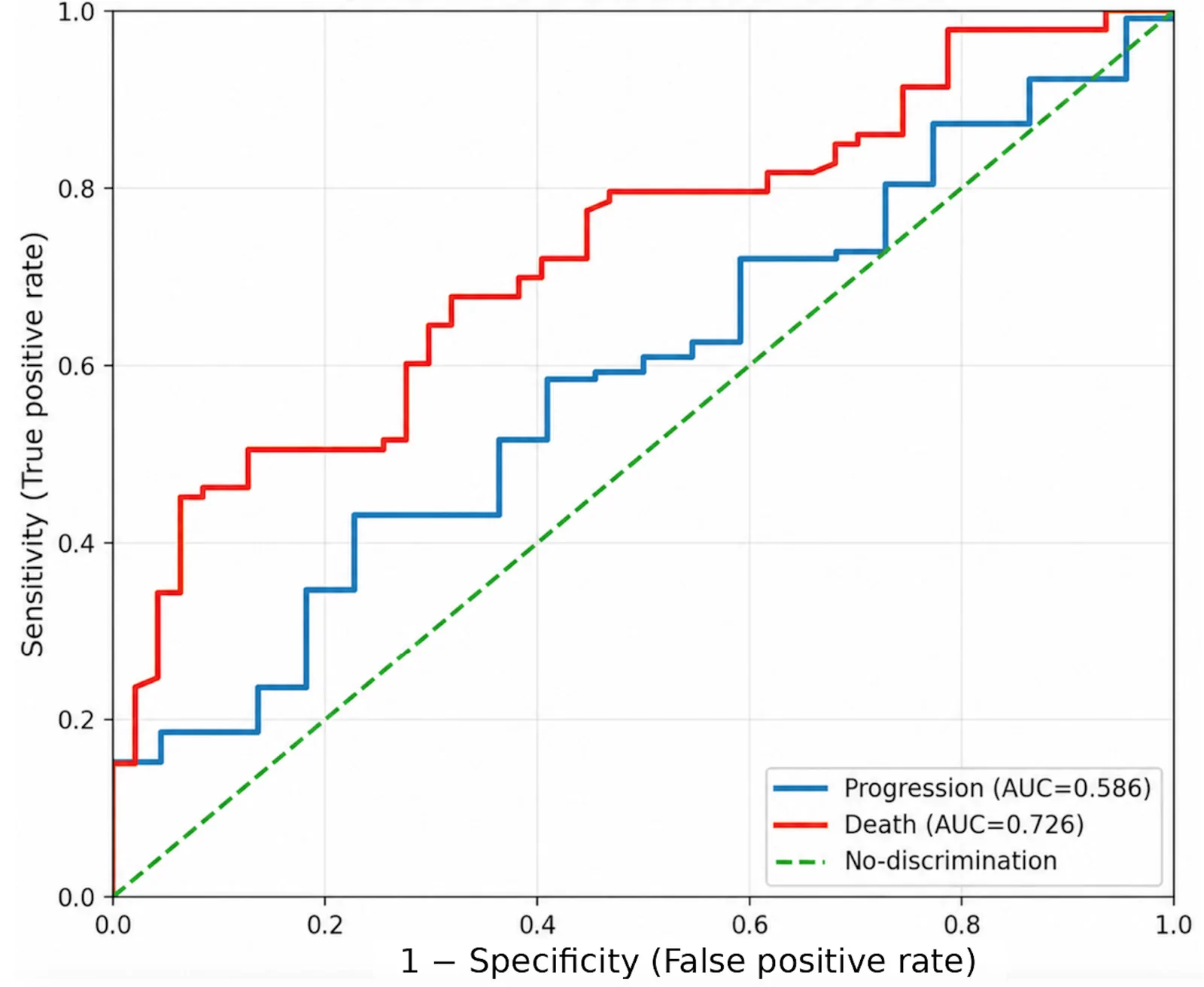
ROC curve analysis of the pan-immune-inflammation value for progression and death.

ROC analysis was performed to determine exploratory PIV cutoff values for progression and death. The ROC curves are shown in Figure 2, and the corresponding values are summarized in Table 2. For progression, the PIV cutoff was 413.3, with an AUC of 0.586, sensitivity of 0.432, and specificity of 0.773. For death, the cutoff was 477.8, with an AUC of 0.726, sensitivity of 0.452, and specificity of 0.936. Thus, the death cutoff showed high specificity but limited sensitivity. The cutoff of 477.8 was used in the exploratory CRS.

The distribution of composite risk score components and risk groups is shown in Table 3.

The composite risk score was calculated using three baseline variables: PR < 20%, visceral metastasis, and PIV ≥ 477.8. Each adverse factor was assigned one point. PR < 20% was present in 46 patients (32.9%) and was more frequent in the palbociclib/letrozole group than in the ribociclib/letrozole group (41.8% vs. 24.6%; *p* = 0.031). Visceral metastasis was present in 66 patients (47.1%) and did not differ significantly between groups (*p* = 0.224). PIV ≥ 477.8 was observed in 46 patients (32.9%), with a higher numerical frequency in the palbociclib/letrozole group, although this difference did not reach statistical significance (40.3% vs. 26.0%; *p* = 0.073).

According to the composite risk score, 35 patients (25.0%) had CRS 0, 57 patients (40.7%) had CRS 1, 43 patients (30.7%) had CRS 2, and 5 patients (3.6%) had CRS 3. Overall, 92 patients (65.7%) were categorized as CRS 0–1, and 48 patients (34.3%) were categorized as CRS 2–3. CRS 2–3 was present in 41.8% of patients in the palbociclib/letrozole group and 27.4% of those in the ribociclib/letrozole group; this difference was not statistically significant (*p* = 0.073).

### 3.3. Survival Outcomes According to Treatment Group

The median follow-up estimated using the reverse Kaplan–Meier method was 52.3 months in the overall cohort, 53.7 months in the palbociclib/letrozole group, and 36.7 months in the ribociclib/letrozole group. A total of 93 deaths were observed, including 46 among patients treated with palbociclib/letrozole and 47 among those treated with ribociclib/letrozole. A total of 118 PFS events occurred, including 58 and 60 events, respectively.

**Figure 3:**
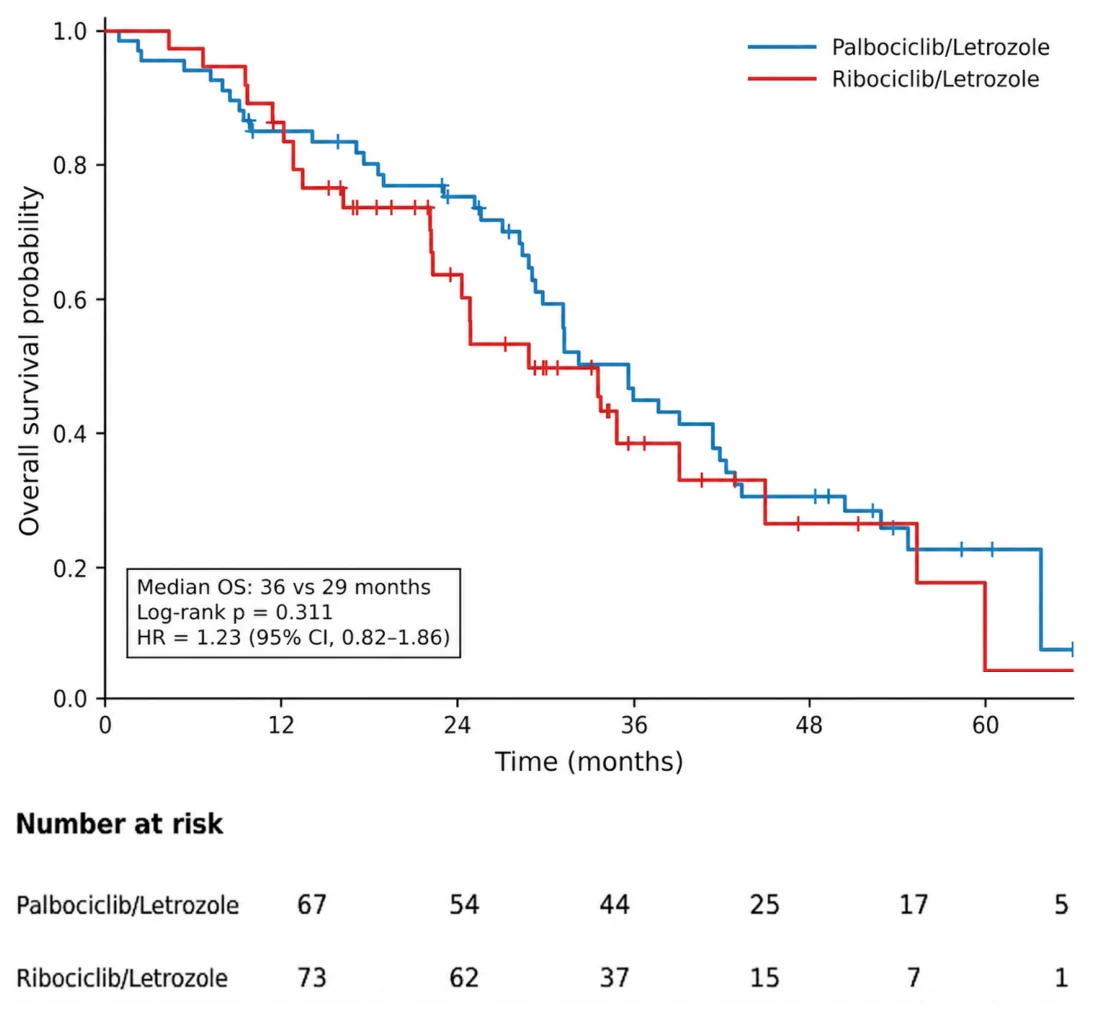
Overall survival according to treatment group.

Overall survival according to treatment group is shown in Figure 3. Median OS was 36 months in the palbociclib/letrozole group and 29 months in the ribociclib/letrozole group. No statistically significant difference was observed by log-rank testing (*p* = 0.311). In univariable Cox regression, ribociclib/letrozole was not significantly associated with OS compared with palbociclib/letrozole (HR, 1.23; 95% CI, 0.82–1.86; *p* = 0.322). Treatment group was also not significantly associated with OS in the parsimonious multivariable clinical model (HR, 1.42; 95% CI, 0.92–2.18; *p* = 0.114). Although the Kaplan–Meier curves crossed visually, no evidence of non-proportional hazards was detected for treatment group (Schoenfeld residual test *p* = 0.914).

**Figure 4:**
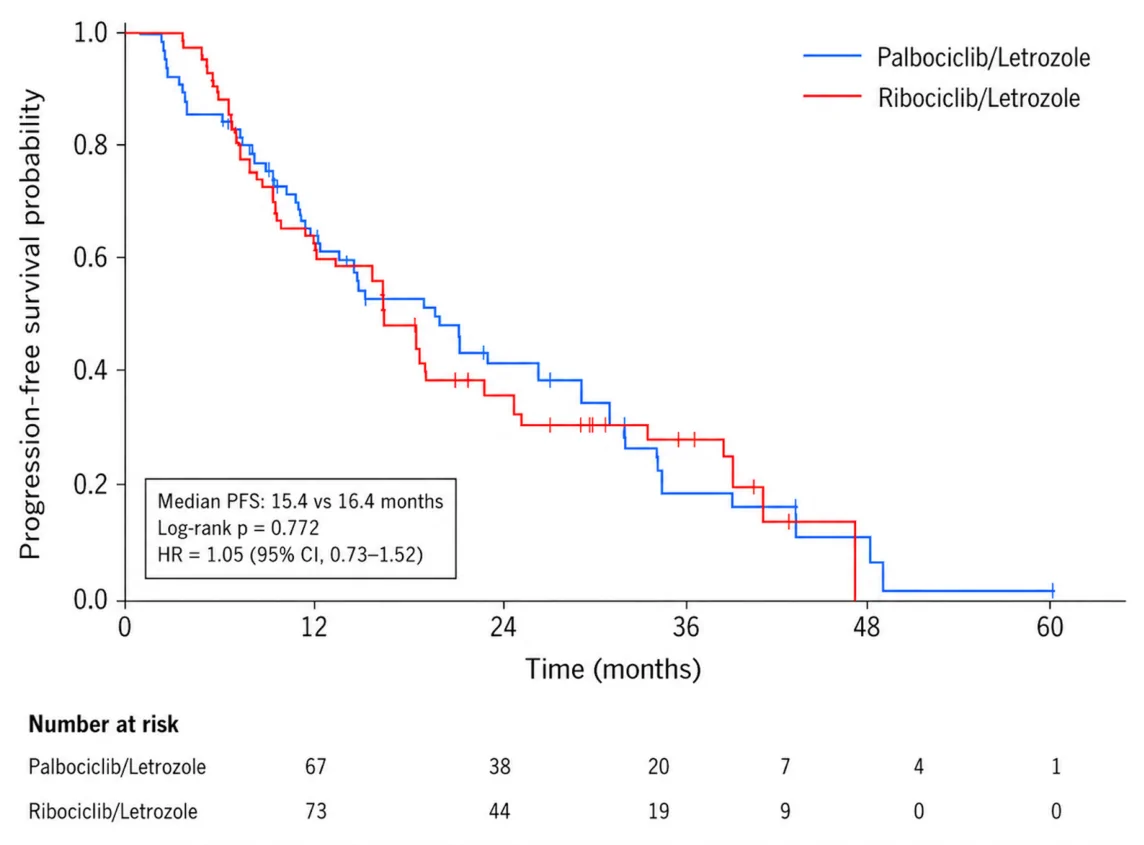
Progression-free survival according to treatment group.

Progression-free survival according to treatment group is shown in Figure 4. Median PFS was 15.4 months in the palbociclib/letrozole group and 16.4 months in the ribociclib/letrozole group. No statistically significant difference was observed by log-rank testing (*p* = 0.772) or Cox regression (HR, 1.05; 95% CI, 0.73–1.52; *p* = 0.778). No evidence of non-proportional hazards was detected in the PFS model (Schoenfeld residual test *p* = 0.743).

### 3.4. Overall Survival According to PIV and Individual CRS Components

Kaplan–Meier and univariable Cox analyses for treatment group, categorical and continuous PIV, visceral metastasis, PR expression, and grouped CRS are summarized in Table 4.

Median OS was 34 months among patients with PIV < 477.8 and 30 months among those with PIV ≥ 477.8. The difference did not reach statistical significance by log-rank testing (*p* = 0.082), and categorical PIV was not significantly associated with OS in univariable Cox analysis (HR, 1.44; 95% CI, 0.94–2.19; *p* = 0.091).

When analyzed as a continuous linear variable, PIV was not significantly associated with OS. The HR per one-standard-deviation increase was 1.02 (95% CI, 0.85–1.22; *p* = 0.850) in the univariable model and 1.05 (95% CI, 0.87–1.27; *p* = 0.593) after adjustment for treatment group. Similarly, the HR per doubling of PIV was 1.01 (95% CI, 0.87–1.18; *p* = 0.873) in the univariable model and 1.05 (95% CI, 0.88–1.24; *p* = 0.598) after adjustment for treatment group.

Visceral metastasis and PR expression < 20% were not significantly associated with OS when evaluated individually. Median OS was 34 months among patients without visceral metastasis and 30 months among those with visceral metastasis (log-rank *p* = 0.211; HR, 1.29; 95% CI, 0.86–1.94; *p* = 0.223). Median OS was 34 months among patients with PR expression ≥ 20% and 32 months among those with PR expression < 20% (log-rank *p* = 0.648; HR, 1.10; 95% CI, 0.72–1.69; *p* = 0.656).

### 3.5. Overall Survival According to Grouped Composite Risk Score

**Figure 5:**
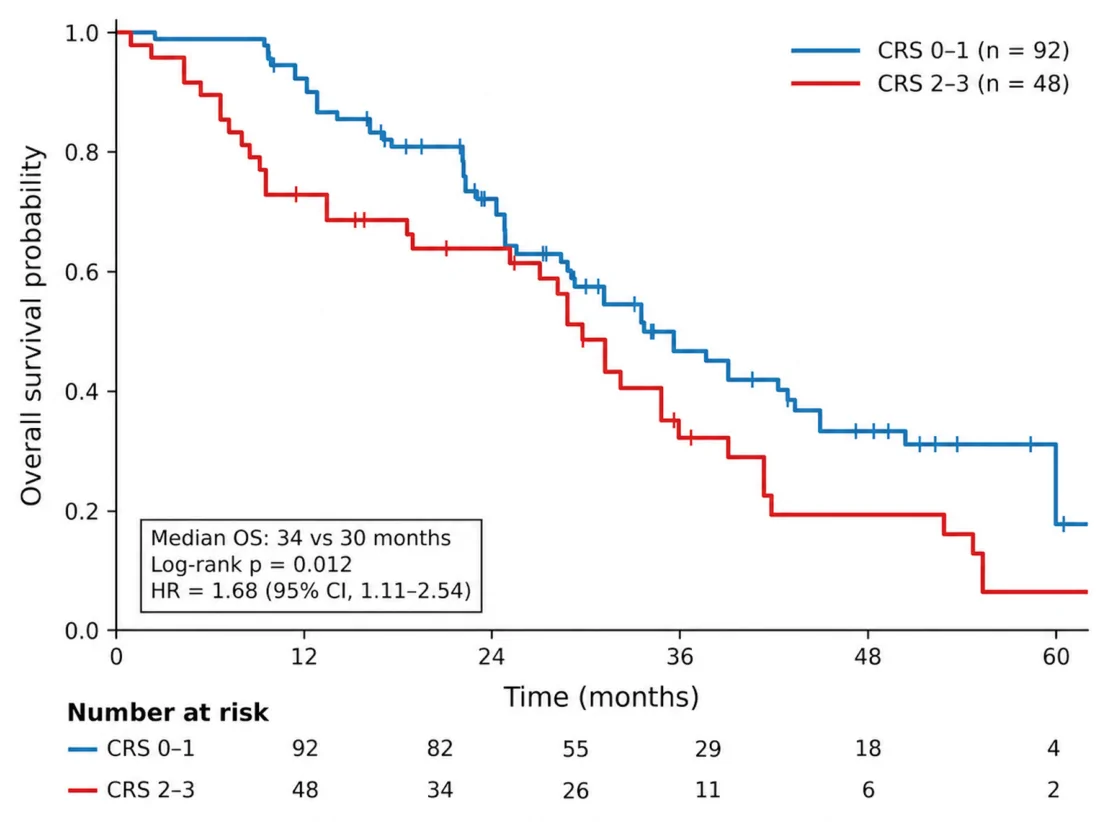
Overall survival according to grouped composite risk score (CRS 0–1 versus CRS 2–3).

Overall survival according to grouped CRS is shown in Figure 5. The grouped CRS survival analysis compared patients with CRS 0–1 (*n* = 92) and those with CRS 2–3 (*n* = 48). Median OS was 34 months in the CRS 0–1 group and 30 months in the CRS 2–3 group. The difference was statistically significant by log-rank testing (*p* = 0.012). CRS 2–3 was associated with a higher hazard of death in univariable Cox analysis (HR, 1.68; 95% CI, 1.11–2.54; *p* = 0.014). No evidence of non-proportional hazards was detected for grouped CRS (Schoenfeld residual test *p* = 0.723). Because only five patients had CRS 3, the grouped comparison was considered more stable than the four-category analysis.

### 3.6. Discriminative Performance and Incremental Prognostic Value of CRS

The discriminative performance and incremental prognostic value of CRS are summarized in Table 5. The apparent Harrell c-index of CRS was 0.573. After bootstrap internal validation, the optimism-corrected c-index was 0.570, indicating limited discrimination. The apparent c-index of the parsimonious clinical model was 0.608 and increased to 0.635 after addition of CRS; the corresponding optimism-corrected values were 0.581 and 0.601. The apparent increase in c-index was 0.027, with a bootstrap interval of −0.005 to 0.077. Addition of CRS did not significantly improve model fit (likelihood-ratio χ^2^ = 2.15; *p* = 0.143). In the expanded model, the HR per one-point increase in CRS was 1.25 (95% CI, 0.93–1.67; *p* = 0.142).

**Table 5:** Discriminative performance and incremental prognostic value of the composite risk score for overall survival.

Model	Apparent c-Index	Optimism-Corrected c-Index	Apparent Δ c-Index (95% Bootstrap Interval)	Likelihood-Ratio Test
CRS alone	0.573	0.570	—	—
Parsimonious clinical model	0.608	0.581	Reference	—
Parsimonious clinical model + CRS	0.635	0.601	0.027 (−0.005 to 0.077)	χ^2^ = 2.15; *p* = 0.143

CRS, composite risk score. The parsimonious clinical model included histologic grade, de novo versus recurrent metastatic presentation, number of metastatic organs, and treatment group. Optimism correction was obtained using bootstrap internal validation. The Δ c-index and likelihood-ratio test compare the model including CRS with the parsimonious clinical model. The reported Δ c-index represents the apparent difference between the two models.

## 4. Discussion

In this real-world cohort, we observed no statistically significant difference in OS or PFS between palbociclib/letrozole and ribociclib/letrozole. CRS 2–3 was associated with shorter OS in univariable analysis, but the score showed limited discrimination and did not improve model fit when added to the parsimonious clinical model. The treatment findings are therefore descriptive, and CRS remains exploratory.

Randomized OS evidence differs across CDK4/6 inhibitor trials. MONALEESA-2 demonstrated a significant OS benefit with ribociclib plus letrozole, whereas the final PALOMA-2 OS analysis did not show a statistically significant OS advantage for palbociclib plus letrozole over letrozole alone [4,5]. However, these findings arose from separate randomized trials with differences in study populations, follow-up, missing data, and post-progression treatment patterns and should not be interpreted as a direct comparison between palbociclib and ribociclib.

The absence of a significant OS benefit in the final PALOMA-2 analysis has not been uniformly mirrored in real-world palbociclib data. DeMichele et al. reported longer real-world PFS and OS with first-line palbociclib plus letrozole compared with letrozole alone, and Rugo et al. reported improved OS and real-world PFS with palbociclib plus an aromatase inhibitor [14,15]. These studies do not provide a direct palbociclib–ribociclib comparison but support the clinical activity of palbociclib-based endocrine therapy in routine practice.

Indirect and real-world comparisons of palbociclib and ribociclib have produced heterogeneous findings. Some analyses have suggested more favorable outcomes with ribociclib, whereas several real-world cohorts have reported no clear survival difference between the agents [8,9,16,17,18]. In the present cohort, treatment allocation was not randomized and was likely influenced by calendar time, physician preference, toxicity considerations, comorbidities, drug interactions, and reimbursement or access conditions. Therefore, the absence of a statistically significant difference should not be interpreted as evidence of treatment equivalence.

Treatment initiation differed substantially by calendar year. Palbociclib was used predominantly during 2020–2021, whereas ribociclib use was concentrated during 2022–2024. Consistently, median follow-up was shorter in the ribociclib group than in the palbociclib group (36.7 vs. 53.7 months), limiting the maturity of late survival estimates in the ribociclib group. Baseline PIV was also higher in the palbociclib group, and the distribution of metastatic organ number differed between groups. These factors prevent a causal comparative-effectiveness interpretation and leave the possibility of residual confounding by indication.

The distribution of best documented response differed between treatment groups. The largest absolute between-group differences were observed in the stable-disease and progressive-disease categories; however, neither ORR nor DCR differed significantly. Moreover, response was retrospectively abstracted from routine-care radiologic and clinical records rather than determined through prospective, protocol-mandated, centrally reviewed RECIST assessment. The response findings should therefore be interpreted descriptively and should not be considered evidence of superior antitumor activity for either regimen.

The grouped CRS analysis showed shorter OS among patients with CRS 2–3 than among those with CRS 0–1. Nevertheless, the optimism-corrected c-index of 0.570 indicated limited discrimination at the individual-patient level. Although addition of CRS to the parsimonious clinical model produced a modest numerical increase in c-index, the bootstrap interval included no improvement, and the likelihood-ratio test was not statistically significant. These findings suggest that CRS may capture cumulative adverse clinical and inflammatory features within this cohort but do not establish that it provides clinically meaningful prognostic information beyond established baseline variables.

Two of the three CRS components, PR expression < 20% and visceral metastasis, were not significantly associated with OS when evaluated individually. Low PR expression remains biologically plausible as a marker of reduced endocrine sensitivity, and visceral involvement is generally associated with adverse prognosis; however, their effects may vary according to disease burden, involved organ, symptoms, and treatment sensitivity [10,11,19]. The present findings should therefore not be interpreted as proof that each component is independently prognostic. Rather, the score may reflect the accumulation of several modest adverse features and may partly be data-driven within this cohort.

PIV showed better ROC discrimination for death than for progression, but the cutoff of 477.8 had a sensitivity of only 45.2%, meaning that it did not identify more than half of the patients who died. It should therefore not be interpreted as a stand-alone clinical decision threshold. In addition, PIV was not significantly associated with OS when modeled as a simple continuous linear variable. PIV is also a nonspecific inflammatory marker and may be affected by infection, corticosteroid exposure, inflammatory comorbidities, bone marrow reserve, and the timing of blood sampling [12,13,20]. Because the cutoff was derived and evaluated in the same cohort, optimism and overfitting remain important concerns.

The median OS estimates in this study were shorter than those reported in pivotal first-line CDK4/6 inhibitor trials. Several real-world factors may have contributed, including metastatic organ burden, visceral involvement, baseline inflammatory status, variation in treatment duration and dose intensity, access to subsequent therapies, comorbidities, and unequal follow-up maturity. Prognosis in de novo metastatic breast cancer may also be influenced by individualized locoregional treatment of the primary tumor in selected patients, although such interventions do not replace effective systemic treatment and their role remains dependent on patient selection [21].

This study has several limitations. Its retrospective, single-center design introduces potential selection bias and limits generalizability. Treatment allocation was not randomized, and residual confounding by indication remains likely despite inclusion of treatment group and selected prognostic factors in a parsimonious clinical model. ECOG performance status was not included in the parsimonious prognostic model; therefore, residual confounding related to performance status cannot be excluded. Treatment initiation years and follow-up maturity differed between groups. Treatment exposure, dose reductions, treatment interruptions, subsequent systemic therapies, and several comorbid or socioeconomic factors were not comprehensively modeled. Response assessment was retrospective and was not based on central RECIST review. PIV was based on a single baseline blood sample, and its stability over time was not assessed. The PIV cutoff and CRS were developed and evaluated in the same cohort, the CRS 3 category included only five patients, and no independent external validation cohort was available. Although bootstrap correction quantified internal optimism, it cannot substitute for external validation.

Taken together, our findings support a descriptive interpretation of the treatment comparison and an exploratory interpretation of CRS; they do not support selecting one CDK4/6 inhibitor over the other or using CRS for individual clinical decisions.

## 5. Conclusions

In this retrospective real-world cohort, no statistically detectable difference in OS or PFS between first-line palbociclib/letrozole and ribociclib/letrozole was observed. Grouped CRS was associated with OS in univariable analysis; however, the score showed limited discrimination and did not significantly improve prognostic performance beyond a parsimonious clinical model. CRS and the ROC-derived PIV cutoff should therefore be regarded as exploratory findings requiring independent external validation.

## Figures and Tables

**Table 1:** Baseline demographic and clinical characteristics and best documented response according to treatment group.

	All Patients (*n* = 140)	Palbociclib/Letrozole (*n* = 67)	Ribociclib/Letrozole (*n* = 73)	*p* Value
**Age (years), median (Q1–Q3)**	60 (55–69)	59 (54–71)	61 (56–66)	0.923
**Age group**				0.205
<65	87 (62.1)	38 (56.7)	49 (67.1)	
≥65	53 (37.9)	29 (43.3)	24 (32.9)	
**ECOG performance status**				0.609
0	73 (52.1)	37 (55.2)	36 (49.3)	
1	62 (44.3)	27 (40.3)	35 (48.0)	
2	5 (3.6)	3 (4.5)	2 (2.7)	
**Progesterone receptor (PR)**				0.088
Negative (<1%)	25 (17.9)	16 (23.9)	9 (12.3)	
Low (1–19%)	21 (15.0)	12 (17.9)	9 (12.3)	
High (≥20%)	94 (67.1)	39 (58.2)	55 (75.4)	
**Ki-67 index (%)**				0.608
<30%	99 (70.7)	46 (68.7)	53 (72.6)	
≥30%	41 (29.3)	21 (31.3)	20 (27.4)	
**Histologic grade**				0.528
1	25 (17.9)	12 (17.9)	13 (17.8)	
2	73 (52.1)	32 (47.8)	41 (56.2)	
3	42 (30.0)	23 (34.3)	19 (26.0)	
**PIV, median (Q1–Q3)**	362.0 (246.8–558.2)	393.1 (267.8–758.4)	328.4 (214.9–475.5)	0.015
**Metastatic presentation**				0.553
De novo	57 (40.7)	29 (43.3)	28 (38.4)	
Recurrent	83 (59.3)	38 (56.7)	45 (61.6)	
**Site of metastases**				
Bone (any)	116 (82.9)	55 (82.1)	61 (83.6)	0.817
Bone (only)	55 (39.3)	31 (46.3)	24 (32.9)	0.105
Distant lymph nodes	46 (32.9)	21 (31.3)	25 (34.2)	0.715
Visceral	66 (47.1)	28 (41.8)	38 (52.1)	0.224
**Number of metastatic organs**				0.046
1	65 (46.4)	37 (55.1)	28 (38.4)	
2	54 (38.6)	18 (26.9)	36 (49.3)	
3	12 (8.6)	6 (9.0)	6 (8.2)	
≥4	9 (6.4)	6 (9.0)	3 (4.1)	
**Treatment initiation year**				<0.001
2020	30 (21.4)	16 (23.9)	14 (19.2)	
2021	37 (26.4)	30 (44.8)	7 (9.6)	
2022	23 (16.4)	8 (11.9)	15 (20.5)	
2023	21 (15.0)	4 (6.0)	17 (23.3)	
2024	24 (17.1)	5 (7.5)	19 (26.0)	
2025	5 (3.6)	4 (6.0)	1 (1.4)	
**Best documented response**				0.029
Complete response	5 (3.6)	3 (4.5)	2 (2.7)	
Partial response	104 (74.3)	52 (77.6)	52 (71.2)	
Stable disease	15 (10.7)	2 (3.0)	13 (17.8)	
Progressive disease	16 (11.4)	10 (14.9)	6 (8.2)	
**ORR**	109 (77.9)	55 (82.1)	54 (73.9)	0.248
**DCR**	124 (88.6)	57 (85.1)	67 (91.8)	0.213

Data are presented as n (%) or median (Q1–Q3). ECOG, Eastern Cooperative Oncology Group; PR, progesterone receptor; PIV, pan-immune-inflammation value; ORR, objective response rate; DCR, disease control rate. Metastatic sites were not mutually exclusive, except for the bone-only category.

**Table 2:** ROC performance of baseline pan-immune-inflammation value for progression and death.

Outcome	AUC	Optimal Cutoff	Sensitivity	Specificity
Progression	0.586	≥413.3	0.432	0.773
Death	0.726	≥477.8	0.452	0.936

AUC, area under the curve. Cutoff values were determined using Youden’s J index. The cutoff of 477.8 was used for the composite risk score.

**Table 3:** Distribution of composite risk score components and score categories according to treatment group.

	All Patients (*n* = 140)	Palbociclib/Letrozole (*n* = 67)	Ribociclib/Letrozole (*n* = 73)	*p* Value
PR expression				0.031
PR < 20% (score 1)	46 (32.9)	28 (41.8)	18 (24.6)	
PR ≥ 20% (score 0)	94 (67.1)	39 (58.2)	55 (75.4)	
**Visceral metastasis**				0.224
Present (score 1)	66 (47.1)	28 (41.8)	38 (52.1)	
Absent (score 0)	74 (52.9)	39 (58.2)	35 (47.9)	
Baseline PIV				0.073
PIV < 477.8 (score 0)	94 (67.1)	40 (59.7)	54 (74.0)	
PIV ≥ 477.8 (score 1)	46 (32.9)	27 (40.3)	19 (26.0)	
CRS category				0.225
0	35 (25.0)	16 (23.9)	19 (26.0)	
1	57 (40.7)	23 (34.3)	34 (46.6)	
2	43 (30.7)	24 (35.8)	19 (26.0)	
3	5 (3.6)	4 (6.0)	1 (1.4)	
Grouped CRS				0.073
CRS 0–1	92 (65.7)	39 (58.2)	53 (72.6)	
CRS 2–3	48 (34.3)	28 (41.8)	20 (27.4)	

Data are presented as n (%). One point was assigned for each of the following factors: PR expression < 20%, visceral metastasis, and PIV ≥ 477.8; the total CRS ranged from 0 to 3. PR, progesterone receptor; PIV, pan-immune-inflammation value; CRS, composite risk score.

**Table 4:** Kaplan–Meier and univariable Cox analyses of overall survival according to treatment group, PIV, individual CRS components, and grouped CRS.

Analysis	Groups/Coding	*n*	Median OS, Months	Log-Rank *p* Value	Univariable Cox HR (95% CI)	*p* Value
**Treatment group**	Palbociclib/letrozole	67	36		Reference	
	Ribociclib/letrozole	73	29	0.311	1.23 (0.82–1.86)	0.322
**Baseline PIV**	<477.8	94	34		Reference	
	≥477.8	46	30	0.082	1.44 (0.94–2.19)	0.091
**Continuous PIV**	Per 1-SD increase	140	—	—	1.02 (0.85–1.22)	0.850
**Log_2_-transformed PIV**	Per doubling	140	—	—	1.01 (0.87–1.18)	0.873
**Visceral metastasis**	Absent	74	34		Reference	
	Present	66	30	0.211	1.29 (0.86–1.94)	0.223
**PR expression**	≥20%	94	34		Reference	
	<20%	46	32	0.648	1.10 (0.72–1.69)	0.656
**Grouped CRS**	CRS 0–1	92	34		Reference	
	CRS 2–3	48	30	0.012	1.68 (1.11–2.54)	0.014

OS, overall survival; HR, hazard ratio; CI, confidence interval; PIV, pan-immune-inflammation value; PR, progesterone receptor; CRS, composite risk score; SD, standard deviation. For categorical comparisons, hazard ratios greater than 1 indicate a higher hazard for the second category listed. Continuous PIV was modeled per one-standard-deviation increase, and log_2_-transformed PIV was modeled per doubling.

## Data Availability

The data supporting the findings of this study are not publicly available due to patient privacy, confidentiality, and ethical restrictions, and cannot be shared in accordance with institutional and ethical approval requirements.
